# The effects of social vs. asocial threats on group cooperation and manipulation of perceived threats

**DOI:** 10.1017/ehs.2020.48

**Published:** 2020-10-12

**Authors:** Pat Barclay, Stephen Benard

**Affiliations:** 1 Department of Psychology, University of Guelph, 50 Stone Rd. E., Guelph, N1G 2W1, ON, Canada; 2 Department of Sociology, Indiana University, Ballantine Hall, 1020 E. Kirkland Ave, Bloomington, 47405, IN, USA

**Keywords:** Social dilemma, public good, threat-dependent cooperation, leadership, intergroup competition, Volunteer's Dilemma

## Abstract

Individuals benefit from maintaining the well-being of their social groups and helping their groups to survive threats such as intergroup competition, harsh environments and epidemics. Correspondingly, much research shows that groups cooperate more when competing against other groups. However, ‘social’ threats (i.e. outgroups) should elicit stronger cooperation than ‘asocial’ threats (e.g. environments, diseases) because (a) social losses involve a competitor's gain and (b) a strong cooperative reaction to defend the group may deter future outgroup threats. We tested this prediction in a multiround public goods game where groups faced periodic risks of failure (i.e. loss of earnings) which could be overcome by sufficient cooperation. This threat of failure was framed as either a social threat (intergroup competition) or an asocial threat (harsh environment). We find that cooperation was higher in response to social threats than asocial threats. We also examined participants’ willingness to manipulate apparent threats to the group: participants raised the perceived threat level similarly for social and asocial threats, but high-ranking participants increased the appearance of social threats more than low-ranking participants did. These results show that people treat social threats differently than asocial threats, and support previous work on leaders’ willingness to manipulate perceived threats.

## Graphical abstract

**Figure:**
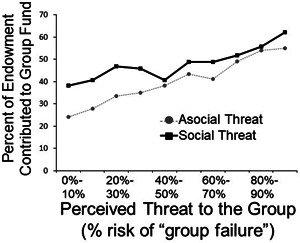

**Media Summary:** Outgroup threats cause more cooperation than asocial threats, esp. if low risk. People manipulate both threats equally

## Introduction

When an external force threatens a group (e.g. a hostile outgroup, natural disaster or epidemic), preventing or overcoming that threat is a cooperative act that benefits all group members. Group members have a stake in each other's welfare because of the benefits of group living (e.g. collective foraging and defence), such that helping the group overcome such threats is individually beneficial (Barclay & Benard, 2013; Kokko et al., 2001; Roberts, 2005). Despite the individual benefits of helping, everyone might prefer that someone *else* expend effort and resources to overcome the group threat. Resolving group threats is thus a Volunteer's Dilemma wherein helping pays when it aids the group to overcome a threat, but not when the threat is absent or inevitable (e.g. Diekmann, 1985, 1986, 1993; Murnighan et al., 1993; Myatt & Wallace, 2008; see Barclay & Van Vugt, 2015, for similar phenomena). Cooperation is more likely in Volunteer's Dilemmas when threats are more likely or costly, such that organisms cooperate when the threat is high and defect when it is low: such ‘threat-dependent cooperation’ has been found in theoretical models (Archetti, 2009), non-humans (Radford, 2008, 2011), and especially in humans (e.g. Barclay & Benard, 2013; Bornstein, 2003; Brewer 2001; Coser 1956; Halevy et al., 2008; Jordan et al., 2017; Lahti & Weinstein, 2005; Milinski et al., 2008; Puurtinen & Mappes, 2009; Sherif, 1966; Van Vugt et al., 2008; Vasi & Macy, 2003; reviewed by Benard & Doan 2011).

Some group members have an incentive to manipulate this tendency to cooperate in the face of group threats. If an organism can manipulate its groupmates into perceiving that the threat is higher than it actually is, then that organism receives higher cooperation from its groupmates, and correspondingly reduced within-group competition (Lahti & Weinstein, 2005; Simmel 1908[1955]; Willer, 2004). Such manipulation could be accomplished by reminders of past threats (e.g. Willer, 2004), ‘us vs. them’ language (Bekkers, 1977), over-responses to ambiguous threats, appearances of constant vigilance or false alarm calls (non-humans: Munn, 1986), or creating actual intergroup conflict (Bekkers, 1977; Dogan et al., 2017). In particular, leaders benefit from manipulating followers’ perceptions of group threats because they receive disproportionate shares of group productivity and have the most to lose from within-group status competition (Dogan et al., 2017; Lahti & Weinstein, 2005). Critics often accuse politicians of threat manipulation, although such charges are difficult to substantiate (e.g. Cinrincione et al., 2004; Tisdale & Norton-Taylor, 2010). Nonetheless, there is anecdotal evidence of threat-manipulation in gangs and other groups (e.g. Brewer 2001; Short & Strodtbeck, 1963) and experimental evidence showing that people – especially those possessing high rank – will initiate intergroup conflict (Dogan et al., 2017) or pay to make group threats appear worse than they actually are (Barclay & Benard, 2013).

Yet what kinds of threats elicit the most cooperation – and the most manipulation? Group threats come in various forms: social threats depend on the actions of others outside the group (e.g. hostile outgroups), whereas asocial threats do not (e.g. natural disasters, predators, diseases, droughts and other ‘games against nature’). Most research does not distinguish between these types of threats (but see Jordan et al., 2017). Organisms should react more strongly to social than asocial threats for at least three reasons. First, other groups are less related to oneself than average (i.e. negative relatedness; Gardner & West, 2004), which fosters spite and gives increased incentives to help related groupmates compete against them (e.g. West & Gardner, 2010; Krupp & Taylor, 2015). Second, defeating an asocial threat means merely preventing a loss whereas defeating a social threat can also involve a gain (e.g. taking an outgroup's resources, land, or mates; Gavrilets, 2015; Manson & Wrangham, 1991) or preventing one's competitors from gaining. Third, social threats are conspecifics who can react to one's group's actions, causing them to mount an even bigger threat in response if they are not deterred outright. A mathematical model by Gavrilets (2015) suggests that group cooperation is more likely to arise first in response to between-group conflicts (social threats) and is then extended to ‘games against nature’ (asocial threats), rather than vice versa.

Based on the reasons above, we predict that humans possess a psychology (e.g. heuristics) that causes them to be more sensitive to social threats than to asocial threats. However, there is little evidence as to whether cooperation is higher in response to social vs. asocial threats. Furthermore, if such a deep-seated psychology exists, it should be elicited even in the absence of actual intergroup competition, just as other deep-seated desires like salivation can be elicited in the absence of actual food (e.g. by saccharine), or sexual arousal can be elicited in the absence of sexual partners (e.g. by pornography). Human behaviour is readily influenced by evolved heuristics (Todd, 2001), including other heuristics about cooperation (e.g. Kioynari et al., 2000), and the framing of a cooperative task can influence which heuristics get activated (Eriksson & Strimling, 2014; Liberman et al., 2004).

As a result, we expect that humans will cooperate to a greater extent when a threat is presented as social in origin, compared with when it is presented as asocial. If people do cooperate more in response to social threats, they may also be more likely to exaggerate the apparent threat when that threat is social in nature, as doing so is more likely to elicit cooperation from group members. Thus, we test whether threat manipulation is more likely when threats are social vs. asocial. We build on past work finding that those occupying high-ranking, contestable positions are more likely to manipulate threats (Barclay & Benard, 2013). We evaluate whether we can replicate this finding in a different setting and sample, and also whether rank moderates the effect of social vs. asocial threats.

The goals of the current manuscript are to test whether people: (a) cooperate more when threats are presented as social vs. asocial; (b) are more responsive to increased levels of social vs. asocial threat; (c) invest more in manipulating group perceptions of social than asocial threats; and (d) are more likely to do each of the above when they possess contestable rank. We use a social vs. asocial framing of an experimental economic game: a modified public goods game with contestable rank, fluctuating threat of group failure (i.e. everyone earns zero) and opportunities to manipulate the threat level that others see. Most importantly, we manipulate the social vs. asocial nature of the threat by the framing alone: the social and asocial threats are identical in structure, incentives and complexity. As such, if participants behave differently to social vs. asocial threats, it is due to the type of threat, rather than some other feature of the situation like the presence of a threshold in social competitions (e.g. Jordan et al., 2017). Laboratory games are simplified models of real-world scenarios: they necessarily lack some features of the real world, like a map that simplifies real terrain and lacks some features of the geography. Nonetheless, such games are useful insofar as they capture essential features of the real world like the conflicting incentives to benefit the group vs. benefit oneself (e.g. Hertwig & Ortmann, 2001; Kokko 2007), and allow us to observe normally unobservable phenomena (e.g. threat manipulation that is typically concealed in real-life settings).

## Methods

### Participants

Participants were 62 males and 58 females from the University of Guelph community (mean age 22.0 ± 5.2 SD years) recruited via posters around campus. Each session included two groups of three participants each; the two groups worked independently and did not interact. As such, there were 40 groups in total, or 120 total participants with 20 rounds each equalling 2,400 total observations. All participants gave written consent before participating. No deception was used: all participants received full instructions and passed a comprehension test before participating. These methods were approved by the Research Ethics Board at the University of Guelph.

### Payment

Participants received CAN$5 for showing up. Participants also earned ‘lab dollars’ (henceforth L$) which were exchangeable to Canadian dollars (CAN$) after the experiment at the pre-announced exchange rate of 3:1, based on the average of all rounds. Total earnings averaged CAN$21.56 (± 4.56 SD).

### Anonymity

Participants’ decisions were made anonymously via computers using z-tree software (Fischbacher, 2007). Communication was not allowed, and partitions prevented visual contact between participants. The experimenter knew participants’ total earnings but not how those earnings were reached.

### Experimental task: modified ‘public goods game’

Participants played 20 rounds of a public goods game in groups of three. Each round, participants received L$50 or L$80 each (see below), and could contribute any whole number of these to a group fund and keep the rest for themselves. The computer program summed the contributions within a group, multiplied them by 1.5, and gave all group members an equal share of the new total (contributors and non-contributors alike). Thus, each L$1 contributed resulted in each group member receiving L$0.50, including the contributor, such that contributions were personally costly but increased the group's earnings.

**Figure 1.**
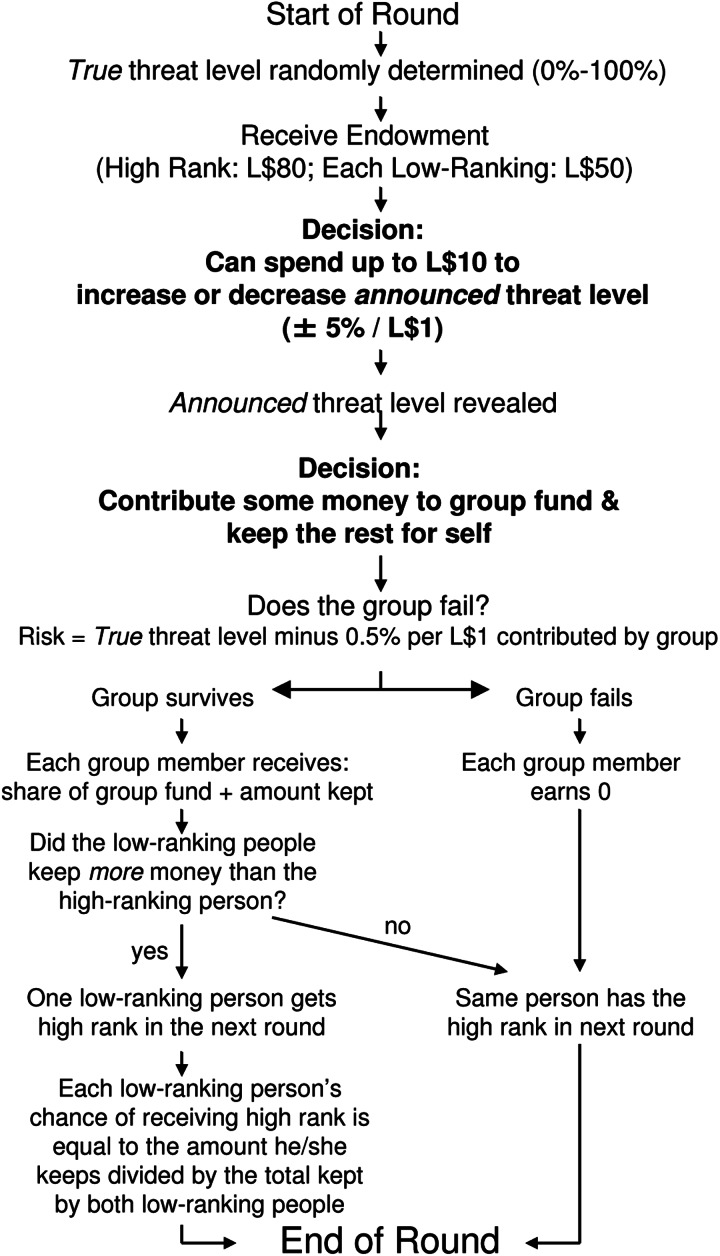
Flowchart of each of the 20 rounds of the study from the perspective of participants. Participants’ decisions are in bold. Threats were framed as the risk of a firm going bankrupt either due to not having enough money to continue operations (asocial threats) or from being outcompeted by other firms (social threats). Figure reused under Creative Commons Attribution (CC BY) license from Barclay and Benard (2013).

In order to test our hypotheses, we modified the public goods game in four ways (based on Barclay & Benard, 2013), which we summarize as follows. First, we included surmountable threats to group survival: each round there was some probability that the group would ‘fail’ and all members would earn zero; participants could reduce this risk by contributing to the public good. Second, we let participants manipulate the apparent severity of those group threats: each participant could pay to increase or decrease the threat level that other participants saw before making their public goods contributions. Thus, participants could exaggerate or downplay the perceived threat level, while the actual threat to the group remained the same. Third, because threat manipulation is more common among high-ranking participants (Barclay & Benard, 2013), we added contestable rank: each group contained one high-ranking member (L$80 endowment) and two low-ranking members (L$50 endowment), and this rank could change each round based on who kept the most money for themselves. Fourth, to test the effects of threat type, we framed the game as either a social threat (a firm being outcompeted by a rival firm) or an asocial threat (a firm going bankrupt from not having enough money to continue operations). In sum, participants engaged in a public goods game, in groups stratified into contestable high- and low-ranking positions, with a fluctuating social or asocial risk of group failure that could be exaggerated or downplayed by participants. We elaborate on each of these four additions in the three sections below. Figure 1 presents an overview of each round.

#### External threats (and manipulation thereof)

To assess how participants respond to (and manipulate) external threats, for each round the computer randomly determined the ‘threat level’. This threat level was the probability that the group would ‘fail’ that round (0–100%, drawn uniformly). If the group failed, then all members earned zero for that round and the previous round, including their endowments and their shares of the group fund. This simulates a group succumbing to external threats, for example by failing to respond effectively to hostile outgroups (e.g. Bowles, 2006), natural disasters or even environmental degradation (Diamond, 2005). Importantly, the threat in the experiment is a continuous probability, which deliberately introduces uncertainty. The uncertain and continuous nature of these threats makes them more ecologically representative of real-life threats than the manipulations used in typical threshold-level public goods and Volunteer's Dilemmas (e.g. Jordan et al., 2017).

The *true* threat level (i.e. the computer-generated risk of failure) was unknown to participants. Instead, they had to rely on the *announced* threat level, which could be manipulated by participants. All group members could pay up to L$10 to increase or decrease what threat level was announced to the group: every L$1 spent this way caused a 5% increase or decrease in the threat level that was announced (but had no effect on the *true* risk of group failure). For example, if the *true* threat level was 40%, and one participant spent L$4 on increasing the threat, then the *announced* threat level would be 60% (i.e. 40 + (4 × 5)%), but the actual risk of group failure would still be 40%. Thus, the sole purpose of increasing or decreasing the *announced* threat level is to change others’ perceptions of the risk of group failure.

After seeing the *announced* threat level, participants decided how much of their L$50 or L$80 endowments to contribute to the group fund. Every L$1 contributed reduced the (*true*) risk of group failure by 0.5%. For example, if the (*true*) threat level was 40% and the group members contributed a total of $20, then at the end of the round there would be a 30% chance (i.e. 40 − (20 × 0.5)%) of the group failing and everyone earning zero in that round. Thus each L$1 contribution to the group fund had two beneficial effects: increasing group earnings by $0.5 per person, and reducing the risk of group failure by 0.5%.

How does this threat level affect the incentives of the game? The threat level adds an element of a Volunteer's Dilemma to the public goods game. If the threat is high enough, then it pays to contribute to the public good to prevent group failure, although all players might prefer that someone else ‘volunteer’ to contribute. This is different from the linear public goods game that many experiments use (a.k.a. *N*-player Prisoner's Dilemma), where defection is the dominant strategy, but the game is still a cooperation game – all public goods fall on a spectrum between a pure Prisoner's Dilemma and a pure Volunteer's Dilemma (Archetti & Scheuring, 2011), and almost all have a mixed equilibrium of cooperation and defection. Threat-dependent cooperation (and manipulation thereof) relies on there being personal benefits from cooperating to overcome the group threat. As such, these external threats – and the Volunteer's Dilemma they create – are a crucial part of our experimental design.

#### Contestable rank

We introduced contestable rank to test whether high and low-ranking participants responded similarly to social and asocial threats, and to see whether they varied in their willingness to manipulate such threats. Previous work showed that high-ranking people spend more to manipulate group threats (Barclay & Benard, 2013), so we used contestable rank to ensure that some participants had sufficient motivation to manipulate group threats, and to recreate the dynamics in real human groups. A diverse range of human social groups – including political parties, labour unions and gangs – are stratified into hierarchies (Gould 2003; Van Vugt et al., 2008). Occupants of high-ranking positions in such groups typically have access to greater resources or power, but also face competition for their position from within the group. There are many definitions of social rank, but common to such definitions are preferential access to resources and the fact that rank is contestable at least in theory (Gould, 2003). The present study used a relatively minimal operationalization of rank – contestable high vs. low endowments each round; previous work examines alternative operationalizations (Barclay & Benard, 2013).

In the present study, the high-ranking participant received L$80 as her endowment each round whereas the low-ranking participants received L$50 each round. This represents preferential access to resources. In any round that the group survived, rank was contestable based on resources: the high-ranking participant lost her position if she kept less money for herself than did the two low-ranking participants combined. After such a supplanting, each low-ranking person's chances of achieving the high rank for the next round depended on their relative amounts kept. For example, suppose that high-ranking A kept L$2 while low-ranking B and C kept $2 and $1, respectively. A would lose the high-ranking position because she kept less (L$2) than B and C combined (L$3); B would get the high rank the next round with 2/3 probability and C with 1/3 probability. We used amounts kept (i.e. not contributed or spent on manipulation) as the basis for rank because we are focusing on hierarchies resembling dominance hierarchies based on raw competitive power (e.g. Henrich & Gil-White, 2001; Reeve & Shen, 2006) as opposed to hierarchies based on prestige and trust (e.g. Barclay, 2004, 2013, 2016; Hardy & Van Vugt, 2006; Milinski et al., 2002; Willer, 2009). In dominance-based hierarchies, helping one's group and competing over rank are generally mutually exclusive, to the point where many authors simply define a failure to cooperate as ‘competition’ (e.g. Kümmerli et al., 2010; Messick & McClintock, 1968; Reeve & Shen, 2006). For a full justification of our definition of rank and its contestability, see Barclay and Benard (2013).

#### Summary of each round

Figure 1 shows the order of events in each of the 20 rounds. First, the computer generated a *true* threat level, i.e. the risk of the group failing and everyone earning zero for that round and the previous one. Participants received their endowments of L$50 (low-rank) or L$80 (high-rank). They could then pay up to L$10 to increase or decrease the *announced* threat level, which affected what others saw as being the threat level but not the actual risk of group failure. After seeing the *announced* threat level, participants could contribute any whole dollar amount to the group fund. Each L$1 contributed reduced the *true* risk of group failure by 0.5%; also, all contributions were summed, multiplied by 1.5 by the computer, and redistributed evenly. If the group failed, everyone earned zero for that round and the previous one, and rank did not change. If the group survived, everyone received their earnings for that round (remaining endowment + share of group fund), and rank was determined by the relative amounts that participants kept. The high-ranking person lost her position if she kept less money than the two low-ranking people; if that happened, then each low-ranking person's probability of winning the high rank depended on their amounts kept relative to each other. To avoid endgame effects (and simulate many real-group life situations), participants did not know the total number of rounds.

#### Experimental conditions: social threats vs. asocial threats

We created social vs. asocial threats by framing the instructions differently across the two conditions. In both conditions, the instructions were framed as three business co-owners choosing to invest their salaries in a ‘company growth account’ (group fund) vs. a ‘personal development account’ (keeping money for self), while competing to be the high-earning ‘sales leader’ (high rank) instead of simply a ‘team member’ (low rank). The threat of group failure was framed as the threat of bankruptcy.

In the Asocial Threat condition (*n =* 60 participants = 20 groups), group failure was framed as going bankrupt (‘franchise failure’) from not having enough money ‘to continue operating effectively’ in an ‘uncertain economy’, or other wording relating to effective operations. In the Social Threat condition (*n =* 60 participants = 20 groups), group failure was framed ‘being out-competed’ from not having enough money ‘to compete effectively with rival businesses’ or other wording relating to outcompeting rival businesses. Thus, the Social Threats were framed much more explicitly as intergroup competition, whereas the Asocial Threats were not. These different framings were repeated multiple times in the instructions, but were still relatively small: the total words that differed between the two framings comprised less than 6% of all words in the introduction and comprehension tests (Asocial Threat, 186/3241 words; Social Threat, 152/3204 words). The Supplementary Information presents both framings, so readers can compare them.

These framings were the only difference between the Asocial Threat and Social Threat conditions; the two conditions were otherwise identical in structure and incentives. As such, any differences between experimental conditions are solely due to the differences in framing (i.e. social vs. asocial threat), as opposed to differences in incentives, complexity or understanding.

#### Analytical strategy

Our data have a multilevel structure in which rounds are nested within individuals, and individuals are nested within groups. The unit of analysis is the person-round; 120 individuals interacting across 20 rounds yields 2,400 total observations. To compensate for the non-independence of observations, we fit multilevel models with random intercepts for individuals and groups, using the *mixed* command in STATA 14.1. Our dependent variables are the percentage of endowments participants (a) contributed to the group and (b) invested in increasing or decreasing the apparent threat level. The threat manipulation measure was coded as a positive number if participants invested in increasing the threat, and a negative number if they invested in decreasing the threat. We focus on percentage of endowment contributed or invested in manipulation in the main text because this measure controls for differences in endowments across participants in high- or low-ranking positions. Our independent variables include rank (high/low), the social/asocial threat manipulation, the interaction of these two variables and controls for perceived threat level and period. Note that our primary independent variable, whether participants are assigned to the Social or Asocial Threat condition, is time-invariant, and so fixed effects models are not appropriate for these data.

To accurately measure each participant's perception of the threat, the control for the perceived threat level was adjusted to take the participant's manipulation of the threat into account. The *perceived* threat level for any given participant is the announced threat level minus however much they changed it by – this accounts for the fact that participants who manipulated the threat would know that some of that threat was not real, i.e. some of the announced threat was caused by them. For example, if the threat announced to the participant on that round was 50%, but the participant had increased the apparent threat by 5%, the *perceived* threat for that participant that round was 45%. Thus, the *true* threat is the probability that the group will fail and is unknown to all participants; the *announced* threat is what was publicly announced, i.e. the true threat plus the sum of everyone's manipulations (including the focal participant); and the *perceived* threat is what any focal participant would perceive after factoring out their own manipulation, i.e. the true threat plus the sum of everyone *else's* manipulations (i.e. excluding the focal participant).

Given that our percentage contribution measure is bounded (ranging from 0–100), one might be concerned about the violation of the assumption that the residuals should be normally distributed. However, the distribution of the residuals, as well as q–q and p–p plots, indicates no signs of violation of this assumption. Because some standardized residuals were large (approximately ± 5), we used robust standard errors, as recommended by Rabe-Hesketh and Skrondall (2012). Comparisons with models without robust standard errors indicated that the presented models are more conservative for most variables (i.e. the presented models generally have larger standard errors).

All *p*-values are two-tailed. In addition to our primary analyses, we fit several alternative models to evaluate the robustness of our results, and to show that they do not depend on one particular modelling strategy or statistical assumptions (see below and Supporting Information). We also conducted checks for influential points, by computing Cook's distance scores for both groups and individuals and comparing them with critical values drawn from an *F* distribution (Aguinis et al., 2013). Depending on the analysis, the significant effects of social threats were either unchanged (i.e. no influential points detected), or had slightly smaller *p*-values compared with those presented in the base models. These results are available from the authors. The data are available on the Open Science Framework at https://osf.io/dvy2j/.

## Results

### Contributions to the group fund

#### Contributions under social vs. asocial threats

**Figure 2.**
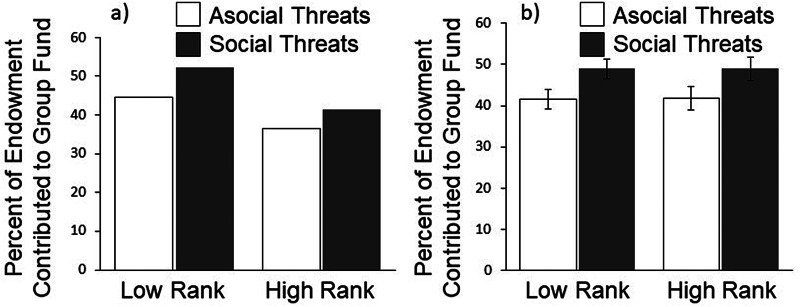
Percentage of endowment contributed to the group fund by high- and low-ranking participants with asocial threats (white bars) vs. social threats (dark bars). Panels show (a) raw means (error bars omitted because raw standard errors are biased due to clustering) and (b) predicted means with cluster-corrected standard errors (which are more conservative).

To test our hypotheses, we present a multilevel model with percentage of endowment contributed to the group fund regressed on rank, the social/asocial threat manipulation, the interaction of these two variables, and controls for perceived threat level and period (see Table 1, column 1). As predicted, participants contributed more to the group fund in the Social Threat condition than the Asocial Threat condition (*b* = 7.36, *p* = 0.029). Figure 2 presents the raw and predicted means from these models, by Social/Asocial Threat condition and rank, with standard errors corrected for clustering; we present both raw and predicted means because error bars for the raw means do not account for clustering and thus underestimate the standard errors. We also conducted a set of supplementary analyses to evaluate the scope and robustness of our findings (see Appendix and Tables S1 and S2 in the Supporting Information for more details). The effects of social threats remained significant and similar in size when employing alternative estimation techniques, including wild cluster bootstrapping (Table S1).

**Table 1.** Multilevel model for the effects (and robust standard errors) of a one unit change (i.e. b-values) in rank, social threats, perceived threat and time period on percentage of endowment spent on contribution and manipulation of perceived threats

	Percentage of endowment contributed	Percentage of endowment spent manipulating the threat level
*Fixed effects*		
High rank	0.179 (1.734)	−0.317 (0.403)
Social threat	7.364* (3.381)	−0.776 (0.598)
High rank × social threat	−0.182 (2.385)	0.952^†^ (0.497)
Perceived threats	0.240** (0.029)	
Period	−0.361** (0.114)	0.032 (0.028)
Constant	32.064** (3.174)	1.541** (0.503)
*Random effects*		
Group-level Random Intercept SD (ln)	1.521* (0.613)	−14.479** (5.442)
Individual-level Random Intercept SD (ln)	2.761** (0.078)	1.259** (0.104)
Observations	2,400	2,400
Number of individuals	120	120
Number of groups	40	40

*Note:* Perceived threats are not included in the manipulation analysis because participants made decisions about manipulating perceived threats before threat levels were announced.

The unit of analysis is the person-round, with person and group-level random intercepts.

^†^*p* < 0.10; *p < 0.05; **p < 0.01.

#### Contributions at different threat levels

**Figure 3.**
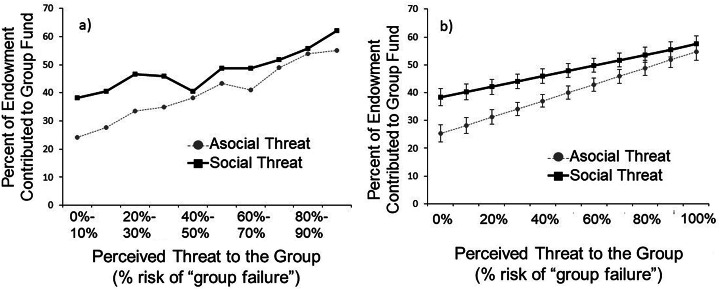
Percentage of endowment contributed to the group fund at different perceived threat levels, for asocial threats (dotted line) and social threats (solid line). Panels show (a) raw means, (error bars omitted because raw standard errors are biased due to clustering) and (b) predicted means with cluster-corrected standard errors.

Participants demonstrated threat-dependent cooperation by contributing more to the group fund when the perceived threat was high: each 1% increase in the perceived threat level was associated with participants contributing an additional 0.24% of their endowment (*b* = 0.24, *p* = 0.0005). In supplementary analyses, we find a marginally significant, negative interaction between social threats and the perceived threat level, such that the difference between social and asocial threats got smaller as the threat level increased (*b* = −0.10, *p* = 0.057; Figure 3, Model 1 in Table S2). While participants consistently contribute more at higher vs. lower threat levels, at low threat levels they contribute more in response to social threats than asocial threats, but at higher threat levels they contribute similarly for social and a social threats.

#### Contributions across periods and by low- and high-ranking participants

We found a negative effect of period, indicating that contributions tend to decline across rounds (*b* = −0.36, *p* = 0.002). Contributions were similar from low- and high-ranking participants: low-ranking participants did not differ from high-ranking participants in their contributions (*b* = 0.18, *p* = 0.918), and rank did not significantly interact with the type of threat (*b* = −0.18, *p* = 0.939). The supplementary analyses also show that high- and low-ranking individuals respond similarly to perceived threats (Models 2 and 3 in Table S2). There is no significant three-way rank × social threat × threat level interaction or subsidiary two-way interactions (Model 3 in Table S2), and the type of threat does not significantly moderate the effect of period (Model 4, Table S2). Across all analyses, the main effect of social threats remains positive and significant.

#### Additional analyses of contributions: group failure and effects thereof

Although not related to our central hypotheses, we conducted several post-hoc analyses on patterns of group failure for interested readers (tables available upon request). Group failure rates were slightly lower in the social threat condition, with a medium effect size (social condition, mean = 0.15 ± 0.07 SD; asocial condition: mean = 0.19 ± 0.09 SD; Cohen's *d* = 0.471). However, because this analysis is at the group level and necessarily has lower power, it was not significant with a *t*-test of the group-level means (*t*_38_ = 1.489, *p* = 0.144) and should be treated with caution.

Did group failure affect participants’ future cooperation? To test this, we fitted models that added two variables to our main model: a lag variable indicating whether the group failed on the previous round, and the interaction between this variable and the social/asocial threat manipulation (Table S2, Model 5). Participants in the asocial threat condition tended to contribute a greater percentage of their endowment to the group when the group failed on a prior round (main effect of group failure: *b* = 7.17, *p* = 0.005); the interaction between group failure and the type of threat was not significant (*b* = −3.56, *p* = 0.304), suggesting that participants in the social threat condition responded similarly to those facing asocial threats.

Our data show that, when the group succeeded, low-ranking participants supplanted the high-ranking person somewhat less often in the social threat condition (i.e. 47% in the social threat condition and 51% in the asocial threat condition). To check whether this difference was statistically significant, we fit a multilevel logit model with random intercepts for group and individual, with whether the high-ranking person was supplanted as the dependent measure and the social/asocial threat condition as the independent measure; the effect of social threat was not significant (table available upon request).

### Manipulation of group threats

#### Overall manipulation

Both high- and low-ranking participants chose to increase the threat level on average rather than lower it: the net manipulation (increases minus decreases) was an overall increase in threats by high-ranking participants in 20/20 rounds (binomial probability *p* = 0.000002) and by low-ranking participants in 19/20 rounds (binomial probability *p* = 0.00004).

#### Manipulation by low- and high-ranking participants

To examine the effects of rank and threat type, we present a multilevel model with percentage of endowment spent increasing the apparent threat regressed on rank, the social/asocial threat manipulation, the interaction of these two variables and controls for period (see Table 1, column 2). Because participants made their manipulation decisions before the threat level was announced, the threat level could not affect these decisions and is not included in the model.

**Figure 4.**
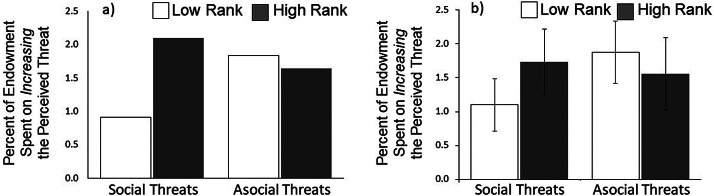
Percentage of endowment spent on *increasing* the perceived threat level by low-ranking (white bars) and high-ranking participants (dark bars). Panels show (a) raw means (error bars omitted because raw standard errors are biased owing to clustering) and (b) predicted means with cluster-corrected standard errors (which are more conservative).

Although we found no significant main effects of rank (*b* = −0.32, *p* = 0.432) or social threats (*b* = −0.78, *p* = 0.194), these findings were qualified by a marginally significant rank × social threat interaction, such that the effect of rank was greater in the social threat condition (*b* = 0.95, *p* = 0.056, Figure 4). We use linear combinations of coefficients to test the pairwise comparison of the effect of high vs. low rank in the social threats condition. With social threats, high-ranking participants spent 0.64% more of their endowments to increase the apparent threat level, compared with low-ranking participants (effect of rank in the social threat condition: *b* = −0.317 + 0.952 = 0.64, *z* = 2.18, *p* = 0.029). In contrast, with asocial threats, high-ranking participants spent a non-significantly different 0.31% less on increasing the threat level, compared with low-ranking participants (as shown by the main effect of rank). This finding was consistent across models using alternative estimation techniques, see Table S3 in the Supplementary Appendix.

#### Cost-effectiveness of manipulation

As in previous work (Barclay & Benard, 2013), manipulation was cost-effective: the L$1.50 that high-ranking participants spent each round on manipulation (across both conditions) resulted in a 7.5% increase in apparent threats, which results in each low-ranking person contributing an additional (7.5 × 0.24) = 1.8% more of their L$50 endowment (i.e. L$0.90 each, L$1.80 total).

#### Additional analysis of manipulation: group failure

Although not related to our central hypotheses, the supplementary analyses show that participants in the asocial threat condition spend less to manipulate group threats when their group failed in the prior round, whereas prior group failure has no effect on participants in the social threat condition (Supplementary Table S2, Supplementary Figure S1).

## Discussion

The results support our main prediction: cooperation is higher when threats are framed as competition with other groups (social threats) than as other types of threats (asocial threats). Participants were more responsive to changes in asocial threats: baseline cooperation was higher for social threats, and this difference diminished and became non-significant at the highest threat levels. Our results also replicate previous experiments showing that people tend to increase rather than decrease the appearance of group threats, especially when they possess high rank, and that they benefit from doing so (Barclay & Benard, 2013). The current results also show that, compared with low-ranking participants, high-ranking participants were likely to manipulate social threats rather than asocial threats, which matches the popular idea of leaders eliciting cooperation via the appearance of social threats like war or terrorism (e.g. Bekkers, 1977; Cinrincione et al., 2004; Lahti & Weinstein, 2005; Tisdale & Norton-Taylor 2010; Willer, 2004) but not asocial threats like natural disasters or pandemics.

In this experiment, the social and asocial threats were identical except for the framing. Thus, our results cannot be explained as participants simply responding to incentives. This allows us to draw conclusions about the underlying psychology and how cooperative the participants were in response to between-group competition, compared with generic threats that do not involve other groups. Our results are suggestive of a psychology that readily reacts strongly to intergroup competition, and this reaction is due to the intergroup nature rather than the incentives or structural aspects of intergroup competition. Our experimental manipulation was relatively small: fewer than 6% of the words differed in the framing of the social and asocial threats. We would predict even bigger effects of threat type if they were real threats, especially if there were kin structure between the groups such that the other groups were negatively related to each actor (e.g. West & Gardner, 2010), or if one's group could garner a reputation that deterred competition from rival groups (Chagnon, 1997; Johnstone & Bshary, 2004; Székely, 2016).

Our results differ from a previous study that compared social vs. asocial threats (Jordan et al., 2017). In that study, participants had to contribute enough money to surpass a given threshold to get a bonus. The threshold was determined either by intergroup competition with another group (i.e. only one group would get the bonus, ‘Competition Threshold’), by the contributions of a previous group whom the participants did not compete with (‘Social Threshold’), or without reference to another group (‘Non-Social Threshold’). Participants contributed more with a threshold than with no threshold, but the type of threshold (social or non-social) did not matter. We speculate that our results differ from their results because we include lower levels of threat, rather than the sharp (and known) threshold used by Jordan and colleagues. Participants in our experiment differentiated between social and asocial threats at low to moderate threat levels, but did not differentiate at high threat levels – dead is dead, and when the threat is very high the source may not matter. High threat levels involve near-certain failure (by definition), which may give people a sufficient incentive to cooperate regardless of the threat type – any additional gains or incentives from strong intergroup competition become superfluous compared with the incentive to avoid near-certain failure. Perhaps if Jordan and colleagues had included less threatening or less sharp thresholds, they may have also found an effect of intergroup competition (i.e. social threats) above and beyond the effects of a threshold alone.

Our results also differ slightly from our previous results. All three studies within Barclay and Benard (2013) found that high-ranking participants manipulate threats more than do low-ranking participants, whereas the current study only found this for social (but not asocial) threats. We have no a priori explanation for this difference, but note that Barclay and Benard (2013) used a neutral description of the game, rather than the business simulation framing employed in the present study. It is possible that the neutral framing of threats in Barclay and Benard (2013) was implicitly interpreted as a threat of between-group conflict. If true, this would be another example of people over-interpreting (negative) events as being caused by agents, and is consistent with many human groups assuming that bad outcomes like disease and crop failure are caused by sorcery or witchcraft from hostile groups (e.g. Boyer, 2002; Chagnon, 1997). Alternately, the difference might reflect differences between the USA (Barclay & Benard, 2013) and Canada (current study) in high-ranking people's willingness to manipulate threats.

We found that high- and low-ranking participants contributed similar percentages of their endowment. It is currently unclear how status, rank or power should relate to cooperation: some theoretical work suggests that high-ranking individuals will contribute more to public goods (Gavrilets & Fortunato, 2014), whereas other work suggests the opposite (Barclay, 2013, 2016; Reeve & Shen, 2006). Some empirical work suggests that people with high status or wealth are more cooperative (e.g. Diekmann, 1993; Diekmann & Przepiorka, 2015; Nettle et al., 2011), whereas other work suggests the opposite (e.g. Barclay & Benard, 2013; Buckley & Croson, 2006; Chan et al., 1997, 1999; Cherry et al., 2005; Guinote et al., 2015; Piff et al., 2012) or finds no effect (Rapoport & Suleiman, 1993; see review by Kafashan et al., 2014). The solution may depend on how different types of cooperation are affected by individual qualities (Barclay & Reeve, 2012), or given that cooperation is risky, on how much different individuals stand to gain from cooperation and are able to buffer against the losses of unreciprocated helping (Barclay et al., 2018; Mishra et al., 2017). Future research is needed on the relationship between rank and cooperation.

### Limitations and future directions

Our study only manipulated the framing of the public goods game, not the actual structure. As such, it lacked the kin structure, possibilities for gain, possibilities of a competitor getting stronger after they win and effects of deterrence that are usually present in intergroup competition. We manipulated the framing in order to examine the underlying psychology rather than how people responded to incentives, but it would be interesting to add some of this complexity back into the game. Future work could also test how the intensity of rivalry with the outgroup affects people's willingness to cooperate with the ingroup to overcome the outgroup threat.

Will our results generalize to other populations (e.g. small-scale non-industrial populations), to other social and asocial threats (e.g. different framings) or to other non-monetary currencies (e.g. investments of time, energy, risk of bodily harm and social status)? Laboratory tasks are abstract models of real-life situations, which capture some important aspects of real life (e.g. conflicting incentives to free-ride vs. protect the group vs. achieve high rank) while stripping away other aspects (e.g. face-to-face interactions, group identity, non-monetary currencies). Laboratory experiments can be helpful when real-life phenomena – such as whether leaders truly perceive an outgroup as a threat vs. a source of political advantage – are difficult or impossible to establish with certainty.

Questions of generalization are always an empirical question. In a classic paper, Mook (1983) argues that the goal of experiments is not to generalize a specific result but to test an underlying theory or principle: a theory predicts that some factor *X* will result in outcome *Y*, so we create an artificial model experiment with factor *X* to see whether we find outcome *Y*. If we do, it supports the theory; if not, then the theory must be revised or rejected (Mook, 1983). So as the quote goes, ‘essentially, all models are wrong, but some models are useful’ (Box & Draper, 1987, p. 424). We argue that whenever the same incentives apply in non-laboratory situations in other societies with other currencies, then we should observe similar effects as were found in this study. The absolute levels of cooperation will undoubtedly differ (e.g. higher overall cooperation in groups with strong identity) and the effect size of social threats may differ too (e.g. stronger effect of social threats if the participants already despise the outgroup), but the general principle will hold. Ultimately though, this is an empirical question, and we look forward to future tests of how different circumstances and cultures affect threat-dependent cooperation and the effects of social vs. asocial threats.

Overall, our study shows that our participants were very sensitive to outgroup competition, more so than to an equivalently sized asocial threat. Furthermore, our results support previous work showing that humans and other animals cooperate more in the face of group threats (Radford, 2011, reviewed by Benard & Doan, 2011) and that high-ranking group members are correspondingly willing to manipulate others’ perceptions of those threats (Barclay & Benard, 2013). We look forward to future work on threat-dependent cooperation, especially mathematical models or computer simulations to identify which factors have the most impact on people's willingness to cooperate in the face of outgroups and to manipulate other's willingness to cooperate.

## Supplementary material

For supplementary material accompanying this paper visit http://dx.doi.org/10.1017/ehs.2020.48. click here to view supplementary material

## Data access

The data and STATA code are available at: https://osf.io/dvy2j/
