# Supplementary material for: The effects of social vs. asocial threats on group cooperation and manipulation of perceived threats
Source: Evol Hum Sci. 2020 Oct 12;2:e54. doi: 10.1017/ehs.2020.48 (PMC10427451; doi:10.1017/ehs.2020.48)
Supplement: Supplementary file 1 [file S2513843X20000481sup001.docx]

Supplementary Material for:

The effects of social versus asocial threats on group cooperation and manipulation of perceived threats

Pat Barclay & Stephen Benard

This Supplementary Material contains:

- Supplementary Analyses of Contributions to the Group Fund: page 2
  - Table S1: page 4
  - Table S2: page 5
- Supplementary Analyses of Manipulation of the Perceived Threat Level: page 7
  - Figure S1: page 8
  - Table S3: page 9
  - Table S4: page 10
- Instructions to Participants: page 11

**Contributions to the Group Fund**

As described in the main text, we conducted a set of supplementary analyses to evaluate the scope and robustness of our findings. We first test whether our results are consistent across different estimation techniques (Table S1). There is currently debate around what constitutes a sufficient number of clusters for multilevel analysis; 30 clusters is often used as a rule of thumb, but various factors can affect the necessary number of clusters. Recommended corrections for small numbers of clusters include using restricted maximum likelihood estimation, using the Kenward-Rogers correction to inflate the standard errors, and bootstrapping (McNeish and Stapleton 2014). In Table S1, we present our original models for comparison, as well as three alternative models implementing these recommendations. The REML and Kenward-Rogers techniques are implemented using STATA’s built-in options for *mixed*. For the bootstrapping analysis, we employ a regression model using multiway clustering using the cgmreg ado-file (Cameron and Miller 2010; .ado file: <http://cameron.econ.ucdavis.edu/research/papers.html>, accessed 2019-12-16); and then bootstrapping coefficients of interest using a wild cluster bootstrap. Wild cluster bootstraps are preferable to other bootstrapping approaches when the number of clusters is relatively small and the regressors include binary variables (Cameron and Miller 2010). The wild cluster bootstrap is implemented using Roodman, Mackinnon, Nielson, and Webb’s (2019) *boottest* postestimation command for STATA. We used the default settings, including 999 replications.

As shown in Table S1, the effects of the social threat variable are consistent across all four estimation strategies, in terms of both substantive size and statistical significance. The only notable difference across the four models is that the multiway clustering model produces a larger and significant negative effect of social rank, such that high-ranking individuals are predicted to contribute a lower proportion of their endowment to the group. We did not obtain this result in any other model specifications. The wild cluster bootstrap of the social threats coefficient produced a bootstrapped confidence interval of [.2121, 16.1], *t*(39) = 2.19, *p* < 0.04. Thus, across all estimation strategies, we find that social threats increase the percentage of endowment invested in contributing to the group, relative to asocial threats.

Next, we evaluate potential moderators by adding additional interaction effects to our model (Table S2). In the first model in this table, we add the social threat x perceived threat interaction, to determine whether social or asocial threats are associated with greater increases in contribution levels. As in the original model, participants contribute more when facing social threats, compared to asocial threats (*b* = 13.23, *p* = .002. This was qualified by a marginally significant, negative interaction between social threats and the perceived threat level, such that the effect of social threats diminished as the threat level increased (*b* = -.10, *p* = .057; raw and predicted means plus cluster-corrected standard errors shown in Figure 3). In other words, participants were more responsive to social threats when the threat level was relatively low, but responded similarly to social and asocial threats when the threat level was high. Linear combinations of these coefficients indicate that the effect of social threats versus asocial threats is significant at the *p* <. 05 level until the threat level is over approximately 60%.^[[1]](#footnote-1)^

In the second model in Table S2, we add the interaction of rank and perceived threat to the main model, to determine whether high or low-ranking individuals respond differently to social or asocial threats. This interaction effect was substantively small and not statistically significant (*b* = 0.004, *p* = .903); we thus find no evidence that rank moderates the effect of perceived threats.

In the third model in Table S2, we add the three-way rank x social threat x threat level interaction (and all subsidiary two-way interactions), as a check on whether the moderating effect of social threats on perceptions of threat is greater or lower by rank; this interaction is not significant (*b* = -.05, *p* = .447). The two-way interaction of social threats and perceptions of threat is no longer marginally significant in this model, due to both a small decrease in the magnitude of the coefficient and a small increase in the standard error.

In the fourth model in Table S2, we added the interaction of period and social/asocial condition to our main model, and found that the type of threat does not significantly moderate the effect of period (*b* = -.31, *p* = .172). Model 5 is described in the main text. Across all analyses, the effect of social threats remains positive and significant.

| \| Table S1: Multilevel model for the effects of Rank, Social Threats, Time Period, and Perceived Threats on Percent of Endowment Spent on Contributing to the Group Fund; Comparing estimation techniques \| \| \| \| \| \| --- \| --- \| --- \| --- \| --- \| \|  \|  \|  \|  \|  \| \| **Fixed Effects** \| ML Estimation  (from main text) \| REML Estimation^2^ \| REML w/  Kenward-Rogers Correction^[[2]](#footnote-2)^ \| Multiway  Clustering \| \| High Rank \| 0.179 \| 0.177 \| 0.177 \| -7.377** \| \|  \| (1.734) \| (1.552) \| (1.552) \| (2.142) \| \| Social Threat \| 7.364* \| 7.364* \| 7.364* \| 7.966* \| \|  \| (3.381) \| (3.534) \| (3.534) \| (3.643) \| \| High Rank x Social Threat \| -0.182 \| -0.182 \| -0.182 \| -1.784 \| \|  \| (2.385) \| (2.220) \| (2.220) \| (3.160) \| \| Perceived Threat \| 0.240** \| 0.240** \| 0.240** \| 0.271** \| \|  \| (0.029) \| (0.017) \| (0.017) \| (0.037) \| \| Period \| -0.361** \| -0.361** \| -0.361** \| -0.362** \| \|  \| (0.114) \| (0.083) \| (0.083) \| (0.116) \| \| Constant \| 32.064** \| 32.069** \| 32.069** \| 32.838** \| \|  \| (3.174) \| (2.816) \| (2.816) \| (3.597) \| \| **Random Effects** \|  \|  \|  \|  \| \| Group-level \| 1.521* \| 1.646** \| 1.646** \| - \| \| Random Intercept SD (ln) \| (0.613) \| (0.579) \| (0.579) \| - \| \| Individual-level \| 2.761** \| 2.761** \| 2.761** \| - \| \| Random Intercept SD (ln) \| (0.078) \| (0.089) \| (0.089) \| - \| \|  \|  \|  \|  \|  \| \| Observations \| 2,400 \| 2,400 \| 2,400 \| 2,400 \| \| Number of individuals \| 120 \| 120 \| 120 \| 120 \| \| Number of groups \| 40 \| 40 \| 40 \| 40 \| \| Note: Robust standard errors in parentheses. Perceived threats are not included in the manipulation analysis because participants made decisions about manipulating perceived threats before threat levels were announced. \| \| \| \|  \| \|  \| \| Multilevel model of proportion of endowment (and standard errors of the coefficients) contributing to the group (column 2). The unit of analysis is the person-round, with person and group-level random intercepts. \| \| \| \|  \| \|  \| \| + *p* < 0.1 \|  \|  \|  \|  \| \| * *p* < 0.05 \|  \|  \|  \|  \| \| ** *p* < 0.01 \|  \|  \|  \|  \|   Table S2: Multilevel model for the effects (and robust standard errors) of Rank, Social Threats, Perceived Threat, Time Period, and Moderating Variables on Percent of Endowment Spent on Contribution | | | | |  |
| --- | --- | --- | --- | --- | --- | --- | --- | --- | --- | --- | --- | --- | --- | --- | --- | --- | --- | --- | --- | --- | --- | --- | --- | --- | --- | --- | --- | --- | --- | --- | --- | --- | --- | --- | --- | --- | --- | --- | --- | --- | --- | --- | --- | --- | --- | --- | --- | --- | --- | --- | --- | --- | --- | --- | --- | --- | --- | --- | --- | --- | --- | --- | --- | --- | --- | --- | --- | --- | --- | --- | --- | --- | --- | --- | --- | --- | --- | --- | --- | --- | --- | --- | --- | --- | --- | --- | --- | --- | --- | --- | --- | --- | --- | --- | --- | --- | --- | --- | --- | --- | --- | --- | --- | --- | --- | --- | --- | --- | --- | --- | --- | --- | --- | --- | --- | --- | --- | --- | --- | --- | --- | --- | --- | --- | --- | --- | --- | --- | --- | --- | --- | --- | --- | --- | --- | --- | --- | --- | --- | --- | --- | --- | --- | --- | --- | --- | --- | --- | --- | --- | --- | --- |
| **Fixed Effects** | Model 1 | Model 2 | Model 3 | Model 4 | Model 5 |
|  |  |  |  |  |  |
| High Rank | 0.298 | -0.030 | -1.147 | 0.179 | -0.193 |
|  | (1.766) | (2.614) | (3.032) | (1.734) | (1.792) |
| Social Threat | 13.232** | 7.363* | 12.407** | 10.570** | 7.795* |
|  | (4.361) | (3.382) | (4.445) | (3.343) | (3.425) |
| High Rank x Social Threat | -0.500 | -0.172 | 1.996 | -0.183 | 0.268 |
|  | (2.404) | (2.386) | (4.370) | (2.386) | (2.554) |
| Perceived Threats | 0.295** | 0.239** | 0.287** | 0.240** | 0.250** |
|  | (0.036) | (0.031) | (0.040) | (0.029) | (0.029) |
| Social Threats x Perceived Threats | -0.104+ |  | -0.090 |  |  |
|  | (0.055) |  | (0.060) |  |  |
| High Rank x Perceived Threats |  | 0.004 | 0.026 |  |  |
|  |  | (0.031) | (0.038) |  |  |
| High Rank x Social Threats x Perceived Threats | |  | -0.046 |  |  |
|  |  |  | (0.061) |  |  |
| Period | -0.365** | -0.361** | -0.365** | -0.208 | -0.369** |
|  | (0.115) | (0.114) | (0.115) | (0.165) | (0.134) |
| Social Threat x Period |  |  |  | -0.305 |  |
|  |  |  |  | (0.224) |  |
| Group Failed Prior Round^1^ |  |  |  |  | 7.170** |
|  |  |  |  |  | (2.559) |
| Group Failed^1^ x Social Threat |  |  |  |  | -3.562 |
|  |  |  |  |  | (3.468) |
| Constant | 28.960** | 32.132** | 29.438** | 30.491** | 30.435** |
|  | (3.365) | (3.272) | (3.493) | (3.178) | (3.193) |
| **Random Effects** |  |  |  |  |  |
| Group-level | 1.498* | 1.522* | 1.499* | 1.521* | 1.639** |
| Random Intercept SD (ln) | (0.638) | (0.612) | (0.639) | (0.613) | (0.517) |
| Individual-level | 2.762** | 2.761** | 2.763** | 2.761** | 2.766** |
| Random Intercept SD (ln) | (0.079) | (0.078) | (0.078) | (0.078) | (0.077) |
|  |  |  |  |  |  |
| Observations | 2,400 | 2,400 | 2,400 | 2,400 | 2,280 |
| Number of individuals | 120 | 120 | 120 | 120 | 120 |
| Number of groups | 40 | 40 | 40 | 40 | 40 |
| The unit of analysis is the person-round, with person and group-level random intercepts. | | | | | |
| ^1^Group failure model has 120 fewer observations because period 1 is excluded, given that lagged failure data are necessarily missing in that round. | | | | | |
| * *p* < .05, ** *p* < .01 |  |  |  |  |  |

**Manipulation of the Perceived Threat Level**

We also conducted supplementary analyses to (1) check the robustness of the effects of social threats on manipulation to different estimation techniques, and (2) check the effects of group failure on manipulation, and whether it moderates the effect of social threats. The findings are similar when comparing across estimation techniques (Table S3). We find no significant effects of rank or social threats in any of the models. Across all models, the interaction of rank and social threats is substantively similar in size, and either significant or marginally significant (*ps* = .034-.056). A wild cluster bootstrapped estimate of the interaction effect of rank and social threat yielded a confidence interval of [-.1092, 2.825], *t*(39) = 1.94, *p* = 0.065).

To examine the effects of group failure on manipulation, we added the lagged effect of group failure in the previous round, and the interaction of group failure and social threats to our main model (see supplementary Table S4). As in the main model, the effects of rank and social threat are not significant, and there is a marginally significant, positive interaction of social threats and rank, such that high-ranking individuals tend to manipulate more in the social threat condition. We also find a significant, negative effect of group failure, indicating that individuals invest a lower percentage of their endowment in manipulation when their group failed on the prior round (*b* = .84, *p* = 0.018). This effect was qualified by a marginally significant, positive interaction of group failure and social threats (*b* = .95, *p* = 0.07), indicating that group failure tended to reduce manipulation in the asocial threat condition, but not the social threat condition (Supplementary Figure S1).


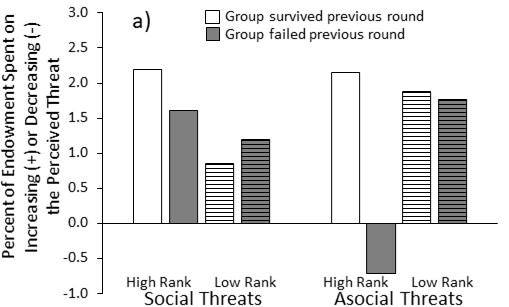

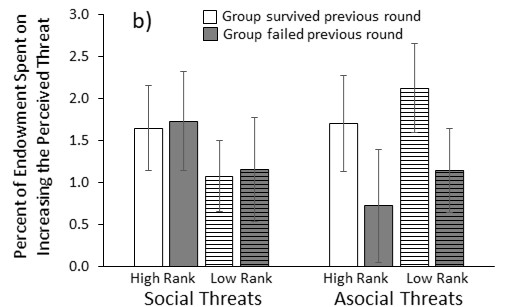


Figure S1: Percent of endowment spent on net increases in the perceived threat level, depending on whether the group survived the previous round (white bars) or failed the previous round (grey bars). High-ranking participants are the unhatched bars, low-ranking participants are the hatched bars. Panels show a) raw means (error bars omitted because raw standard errors are biased due to clustering); b) estimated means with cluster-corrected standard errors.

| Table S3: Multilevel model for the effects of Rank, Social Threats, Time Period, and Group Failure on Percent of Endowment Spent on Manipulating the Threat Level; Comparing estimation techniques | | | | |  |
| --- | --- | --- | --- | --- | --- |
| **Fixed Effects** | ML Estimation  (from main text) | REML Estimation | REML  w/Kenward-Rogers Correction | Multiway Clustering |  |
| High Rank | -0.317 | -0.317 | -0.317 | -0.189 |  |
|  | (0.403) | (0.314) | (0.314) | (0.589) |  |
| Social Threat | -0.776 | -0.776 | -0.776 | -0.918 |  |
|  | (0.598) | (0.693) | (0.693) | (0.644) |  |
| High Rank x Social Threat | 0.952+ | 0.951* | 0.951* | 1.377+ |  |
|  | (0.497) | (0.449) | (0.449) | (0.709) |  |
| Period | 0.032 | 0.032+ | 0.032+ | 0.032 |  |
|  | (0.028) | (0.017) | (0.017) | (0.028) |  |
| Constant | 1.541** | 1.541** | 1.541** | 1.498** |  |
|  | (0.503) | (0.520) | (0.520) | (0.553) |  |
| **Random Effects** |  |  |  |  |  |
| Group-level | -14.479** | -14.973 | -14.973 | - |  |
| Random Intercept SD (ln) | (5.442) | (788.647) | (788.647) | - |  |
| Individual-level | 1.259** | 1.269** | 1.269** | - |  |
| Random Intercept SD (ln) | (0.104) | (0.071) | (0.071) | - |  |
|  |  |  |  |  |  |
| Observations | 2,400 | 2,400 | 2,400 | 2,400 |  |
| Number of individuals | 120 | 120 | 120 | 120 |  |
| Number of groups | 40 | 40 | 40 | 40 |  |
| Note: Robust standard errors in parentheses. Perceived threats are not included in the manipulation analysis because participants made decisions about manipulating perceived threats before threat levels were announced. | | | | | |
| Multilevel model of proportion of endowment (and standard errors of the coefficients) spent on increasing the threat level (column 1) and contributing to the group (column 2) in Study 1. The unit of analysis is the person-round, with person and group-level random intercepts. | | | | | |
| + *p* < 0.1 |  |  |  |  |  |
| * *p* < 0.05 |  |  |  |  |  |
| ** *p* < 0.01 |  |  |  |  |  |

| Table S4: Multilevel model for the effects (and Robust Standard Errors) of Rank, Social Threats, Time Period, and Group Failure on the Percent of Endowment Spent on Manipulating the Threat Level | |
| --- | --- |
|  | Percent of Endowment |
| **Fixed Effects** | Spent Manipulating the Threat Level |
| High Rank | -0.383 |
|  | (0.436) |
| Social Threat | -0.984 |
|  | (0.619) |
| High Rank x Social Threat | 1.039+ |
|  | (0.550) |
|  |  |
|  |  |
| Period | 0.040 |
|  | (0.028) |
| Group Failed Prior Round | -0.843* |
|  | (0.357) |
| Group Failed x Social Threat | 0.945+ |
|  | (0.522) |
| Constant | 1.620** |
|  | (0.523) |
| **Random Effects** |  |
| Group-level | -15.711 |
| Random Intercept SD (ln) | (31.465) |
| Individual-level | 1.274** |
| Random Intercept SD (ln) | (0.107) |
|  |  |
| Observations | 2,280 |
| Number of individuals | 120 |
| Number of groups | 40 |
| Notes: Perceived threats are not included in the manipulation analysis because participants made decisions about manipulating perceived threats before threat levels were announced. The unit of analysis is the person-round, with person and group-level random intercepts. Group failure model has 120 fewer observations because period 1 is excluded, given that lagged failure data is necessarily missing that round. | |
| * *p* < 0.05 |  |
| ** *p* < 0.01 |  |

**Instructions for Participants**

Participants read the instructions on a series of screens, and had a comprehension test at the end. If a participants answered a question incorrectly on the comprehension test, then the program told them that their response was incorrect, told them to ask the experimenter for clarification, and did not continue until the participant entered the correct response. This ensured that participants could not do the experiment until they had answered all questions correctly.

Here we present the text from these screens, with the differences between the experimental conditions presented in bold **[asocial threats / social threats]**.

Slide 1

Welcome to the decision-making experiment. This program explains how the experiment works, and then you will do the task and earn money for your decisions. Your exact payoffs will depend upon your decisions and those of the other participants. We will collect your decisions without knowing what any person decided to do, so that all of your choices will be anonymous.

Please go through these instructions at your own pace. If you are confused, please feel free to ask questions. However, please do not ask questions like “What is the BEST decision to make?” or “What should I do to make lots of money?”. You are free to make whatever decisions you would like. The experimenter can help you to understand the task, but cannot advice you about what decisions are “best”.

Slide 2 – Basics

The following experiment constitutes a simulated business situation in which you will make business-style decisions modelled on the world of finance and economics in order to grow your business and maximize your profits.

You have decided to put your business skills to the test and purchase a franchise with two other associates. You will interact with these people for the entire session. You will not know which other people are in your group. You must work together to ensure the success of your business. Failure to do so will result in **[an inability to respond to a fluctuating economy and being driven out of the market / being competed out of the market by a previously established rival franchise]**, forcing a declaration of bankruptcy and a loss of all the money invested by yourself and your associates.

Your exact payoffs will depend upon your decisions and the decisions of the others in your group. You will do the task for a number of rounds, and you will be paid based on the average of all rounds.

Slide 3 – Group Task

The experiment is divided into a number of rounds. In each round, each franchise co-owner will start with a salary of either 50 or 80 lab dollars. In each round, you can either invest these dollars into a company growth account or into a personal development account. These decisions will affect your franchise’s ability to **[continue operating effectively under a fluctuating economy in order to avoid bankruptcy / compete effectively with previously established rival businesses in order to avoid being driven out of the market and going bankrupt]**.

All money invested in the company growth account will be summed up across all franchise co-owners, and will go towards financing advertising, merchandising, company promotions, marketing research, and other business-directed investments. This will improve the strength of your franchise and the likelihood of the business **[surviving through an uncertain economy / being able to compete with the rival business]** and avoiding bankruptcy. All money invested in the company growth account will reliably return a profit of 1.5 times the initial investment. In other words, the franchise co-owners earn 1.5 times as much as the total investment in the company growth account. That new total will be divided evenly amongst all franchise co-owners.

For example, if everyone contributes $50, there will be $150 total in the company growth account, which gets multiplied by 1.5 to become $225, so that everyone receives a $75 share. This will also help the company **[keep operating despite changes in the economic market / compete with the rival business]** (this will be explained in detail later).

On the other hand, if no one contributes anything, then there is no money on which a return can be made, so each person only earns the money that he/she started with. In this case, there will be no money in the company growth account, and your business will later find it very difficult to **[survive the fluctuating economy / compete with the rival business]**.

Thus, you can earn more money and enable your franchise to **[prosper and grow while avoiding bankruptcy / compete with the rival business]** when people invest in the company growth account.

Slide 4 – Individual Earnings

However, each individual keeps for him/herself any money that he/she invested in the personal development account. Every dollar in your personal investment account means you privately earn that dollar (i.e. it is not divided or shared).

All of you will receive equal shares of the company’s earnings, whether or not you invest in the company growth account.

In addition to your private earnings, money invested in the personal development account contributes to financing personal training programs, development courses, self-promotion within the company, and other self-directed investments which improve your individual performance and increase the likelihood of making the move from being a Junior Partner (low rank) to a Senior Partner (high-rank) within the company (this will be explained later).

Thus, although all co-owners would do well if everyone invests in the company growth account, you can try to make more money for yourself by investing in the personal development account. Please note that every dollar you invest in your personal development account means one less dollar you can invest in the company growth account to help the company **[handle the fluctuating economy / outcompete the rival business]** and avoid bankruptcy.

If you have any questions about the task, please ask them now.

Slide 5 – Examples of Payoffs

For the sake of simplicity in these examples, imagine that everyone starts with a salary of $50 (i.e. no one has $80).

Example 1. If the other members invest $50 each ($100 total) in the company growth account, and you invest $0 (i.e. you invest all $50 in your personal development account). The total investment in the company growth account would be $100. The company earns $150 and you get a $50 share of that.

In this example, you earn the $50 in your personal development account plus your $50 share of the company’s earnings, for a total of $100, whereas the others would only earn the $50 share (because they invested everything in the company growth account).

Example 2. If you invest $50 in the company growth account (and nothing in your personal development account) and the other two people invest $0, then the total investment in the company growth account is $50. The company earns $75, and you get a $25 share of that.

In this example, you only earn the $25 share of the company’s earnings, whereas the other members also earn the $50 they each invested in their personal development account, for a total of $75 each.

If you have any questions about these examples, please ask them now.

Slide 6 – Rank

There are two roles within the company, high-ranked Senior Partners and low-ranked Junior Partners.

The person in the high-ranked Senior Partner role will start each round with a salary of $80, and those with the low-ranked Junior Partner role will start each round with a salary of $50. Senior Partners thus have more money that they can invest in the company growth account (to increase franchise earnings and help the company to **[prevent bankruptcy due to the fluctuating economy / outcompete the rival business]**) or in the personal development account (to increase their potential earnings and the likelihood of maintaining the high rank).

These ranks are not fixed: the Senior Partner will lose this position if the two Junior Partners invest more money combined in their personal development accounts in any given round than the high-ranked Senior Partner does.

In other words, if the total amount of money in the Junior Partners’ personal development accounts (together) is higher than the amount of money in the Senior Partner’s personal development account (alone), then one of the low-ranked Junior Partners will win the high-ranked Senior Partner role for the following round.

In the event of such a switch, a Junior Partner’s chance of winning the Senior Partner role depends on his/her personal investment relative to the other low-ranked Junior Partner: e.g. if one of them invests twice as much in the personal development account as the other, then he/she is twice as likely to win the Senior Partner role.

Ranks will only change if the company succeeds **[in keeping the business operating in a fluctuating economy / at outcompeting the rival business]** by investing enough funds in the company growth account. If **[the franchise fails and declares bankruptcy / goes bankrupt because rival business wins the competition]**, then the ranks will not change in the following round.

If you have any questions about these ranks, please ask now.

Slide 7 – Examples of Changes in Rank

Example 1: Suppose that the Senior Partner invests $40 in his/her personal development account, and the two Junior Partners invest $10 and $20, respectively. In this case, the Senior Partner keeps his/her rank for the following round because he/she invested more in his/her personal development account than the two Junior Partners combined ($30).

Example 2: Instead, suppose that the Senior Partner invests $25 in his/her personal development account and the two Junior Partners invest $10 and $20, respectively. In this case, the Senior Partner loses his/her position because the Junior Partner’s combined personal investments are higher ($30). One of the two Junior Partners will win the high rank for the following round – the first Junior Partner has 1/3 chance [(10/(10+20) = 10/30 = 1/3] of getting this position and the second Junior Partner has 2/3 chance [20/(10+20) = 20/30 = 2/3]

Example 3: Instead, suppose that the Senior Partner invests $40 in his/her personal development account and the two Junior Partners invest $30 and $20. In this case, the Senior Partner loses his/her position because the Junior Partners’ combined personal investments are higher ($50). One of the two Junior Partners will win the Senior Partner role for the following round – the first Junior Partner has a 3/5 (%60) [30/(30+20) = 30/50 = 3/5] of gaining this position and the second Junior Partner has a 2/5 (%40) chance [20/(30+20) = 20/50 = 2/5].

If the Senior Partner’s personal investment is exactly the same as the two Junior Partners combined, then he/she keeps the high rank. The two Junior Partners’ combined personal investment must be GREATER than the Senior Partner’s in order to cause a change in rank.

If you have any questions about these changes in rank, please ask them now.

Slide 8 – Group Failure

**[Every round, the economy can move up or down. Because of the fluctuating economy, there will be a chance that the franchise will be unable to generate enough of an investment from its co-owners to be able to continue operating, resulting in the failure of your franchise and a forced declaration of bankruptcy. / Every round, there will be a chance that the franchise will be out-competed in the market by a previously established rival business, resulting in the failure of your franchise and a forced declaration of bankruptcy.]** If this happens, all franchise co-owners earn no money in that round OR IN THE PREVIOUS ROUND. In other words, franchise failure means you lose the money earned in the current AND the previous round... because you are paying off company debts. This can have a major effect on your earnings.

**[Based on the state of the economy each round / Based on competition from a rival business]**, the threat of bankruptcy is a percentage that will vary from round to round. When the threat level is low, **[the investment required from the co-owners will be minimal and there will be a low probability that the franchise will fail / there will be a low probability that the rival business will be strong enough to drive you out of the market]**, and when the threat level is high, **[greater investments from the co-owners will be necessary to avoid bankruptcy because there will otherwise be a high probability that the franchise will fail / there will be a high probability that the rival business will be able to force you to declare bankruptcy]**.

Every dollar invested in the company growth account decreases the threat of bankruptcy by 0.5%, such that the franchise is less likely to be **[defeated by an uncertain economy / out-competed by the rival business]** if more is invested in the company growth account. If contributions are high enough, they can reduce the threat of bankruptcy to 0% in almost any round.

Because a franchise failure and bankruptcy causes all co-owners to earn zero for two rounds and the ranks will not change, it is in all franchise members’ interest to ensure that the franchise does not fail.

If you have any questions about this task, please ask them now.

Slide 9 – Altering the Threat Level

The threat level posed by **[a fluctuating economy / the rival business]** will be determined by the computer at the start of each round. Before anyone finds out what level it is, all company members will have the option to pay to change the level that is presented to their two associates.

It costs one dollar to increase or decrease the perceived threat level by five percent, and you can spend up to ten dollars on this per round.

Please note: this does not change the actual threat level, but it does change what other company members SEE as being the threat level. The only thing that changes that actual threat posed by **[an uncertain economic environment / the rival business]** is investment in the company growth account.

People can pay different amounts to change the perceived threat level – all changes will be summed up across all company members, and everyone will only see the threat level after all changes have been applied. After seeing this threat level, people will make their decisions about investing in the company growth account or personal development account

Please note that although people can change the perceived threat level slightly, the actual threat level can vary greatly between rounds due to **[a fluctuating economy / varying degrees of competition posed by the rival business]** such that it could be quite high in some rounds and quite low (or zero) in others.

If you have any questions about this part of the task, please ask them now.

Slide 10 – Recap of the experiment

You will operating a new company with two other associates, and you will be making decisions regarding how best to ensure that the company has enough funds to **[continue operating in an uncertain economy to avoid bankruptcy / avoid bankruptcy by outcompeting a rival company]**, while maximizing your own role in the franchise and thereby increasing your personal earnings.

You will do a number of rounds, and will be paid based on the average of these rounds. Every round, you will receive a salary of either $50 (if you are the low-ranked Junior Partner) or $80 (if you are the high-ranked Senior Partner), and you must divide this between the company growth account and your personal development account.

All money in the company growth account goes towards business-directed investments which improve the likelihood of the business prospering and being able to continue having the required funds to **[operate the franchise under a fluctuating economy while avoiding bankruptcy / outcompete the rival business**. Money invested in this account reliably produces a return of 1.5 times the initial investment, and all franchise members receive an equal share of this new total regardless of their investment in the company growth account. Any investments in your personal development account are kept personally by you.

If the two Junior Partners’ combined personal investments are higher in any given round than those of the Senior Partner, then the Senior Partner loses this position, and one of the two Junior Partners wins it for the next round.

Every round, there is a chance that the group will be unable to raise enough funds to **[survive in an uncertain economic environment / outcompete the rival business]** and will be forced to declare bankruptcy, with no one earning any money if this occurs. Every dollar invested in the company growth account decreases this threat level by 0.5%.

Any franchise member can pay $1 to increase or decrease the PERCEIVED threat level by 5% (can spend up to $10). This does not change the actual threat level, but it does change what other franchise members see as being the threat level.

If you have any questions about this task, please ask them now.

Slide 11 – Quiz

1a) If the total investment in the company growth account is $100, what is the new total in the company growth account that will be distributed across all franchise members?

1b) What would be your individual share of that amount be? (remember that there are three people per franchise)

1c) In this example, if you had invested $25 in your personal development account, what would your total earnings be?

2a) If the total investment in the company growth account is $20, what is the new total in the company growth account that will be distributed across all franchise members?

2b) What would your individual share of that amount be? (again, three people per franchise)

2c) In this example, if you had invested $40 in your personal development account, what would your earnings be?

3) If the Senior Partner invests $39 in his/her personal development accounts, what would the combined personal investments of the two Junior Partners have to be in order to cause a change in rank? (In other words, they have to be AT LEAST this much… this is the MINIMUM mount that would cause a change in rank)

4) Imagine there is a change in rank. If one Junior Partner invests $4 in his/her personal development account while the other invests $6, how likely is it (in percent i.e. out of 100) that the second Junior Partner will win the Senior Partner role for the following round?

True/False: the Senior Partner in each round starts out with $80, whereas the two Junior Partners start with only $50 each.

True/False: if you pay to alter the announced threat level, then it directly reduces the likelihood of franchise being **[unable to generate enough investment from the co-owners to continue operating under and uncertain economy / out-competed by a rival business]** in that round.

True/False: if the franchise **[fails to generate enough of an investment from the co-owners / is out-competed by a rival business]**, then you lose more than one round’s worth of earnings.

True/False: you will be paid based on the average of rounds

Slide 12 – Final Details

Before doing the task for real, you will do one practice round to get used to the program. This practice round is identical to the paid rounds, except that it will not count towards your study pay. If you have questions during or immediately after this round, please ask the experimenter. It will be clear when the practice rounds end and when the paid rounds start.

Please remember that once the paid rounds start, your study pay will be based on the average of all rounds, so you will want to treat each round seriously.

The experimenter will need to know your earnings in order to pay you, but he/she will not know any of your actual decisions. Your earnings will be kept confidential (no one but the experimenter will know), and your decisions will be anonymous (no one, including the experimenter, will know). Please DO NOT reveal your decisions to other participants, as you will lose your anonymity if you do so.

To preserve anonymity, we ask that you refrain from talking to other participants from this point on. If you are confused, or if you have any questions, please ask the experimenter. However, please do not ask questions like “What is the best decision to make?” or “What should I do if I want to make lots or money?” because you will be free to make whatever decisions you want once the decision-making tasks start. If you talk or communicate with other participants during the tasks, you may be excluded from the task and from any study pay.

If you have any questions about these details, please ask them now.

1. Calculated using STATA’s *lincom* command, examining the effect of social threats when the threat level ranges from 0 to 100 in intervals of 10 (e.g., for the 50% level, 13.231 – 50*.104 = *b* =8.06, *p* = 0.017) [↑](#footnote-ref-1)
2. The results of the REML with Kenward-Rogers Correction appear to be identical to the results without this correction, but this is due to rounding. In other words, the two analyses produce slightly different results which round to the same values. [↑](#footnote-ref-2)
